# Modulation frequency selection and efficient look-up table inversion for frequency domain diffuse optical spectroscopy

**DOI:** 10.1117/1.JBO.26.3.036007

**Published:** 2021-03-25

**Authors:** Matthew B. Applegate, Carlos A. Gómez, Darren Roblyer

**Affiliations:** Boston University, Department of Biomedical Engineering, Boston, Massachusetts, United States

**Keywords:** diffuse optics, frequency domain, modulation frequency, noise model, high speed

## Abstract

**Significance:** Frequency domain diffuse optical spectroscopy (FD-DOS) uses intensity modulated light to measure the absorption and reduced scattering coefficients of turbid media such as biological tissue. Some FD-DOS instruments utilize a single modulation frequency, whereas others use hundreds of frequencies. The effect of modulation frequency choice and measurement bandwidth on optical property (OP) extraction accuracy has not yet been fully characterized.

**Aim:** We aim to assess the effect of modulation frequency selection on OP extraction error and develop a high-speed look-up table (LUT) approach for OP estimation.

**Approach:** We first used noise-free simulations of light transport in homogeneous media to determine optimized iterative inversion model parameters and developed a new multi-frequency LUT method to increase the speed of inversion. We then used experimentally derived noise models for two FD-DOS instruments to generate realistic simulated data for a broad range of OPs and modulation frequencies to test OP extraction accuracy.

**Results:** We found that repeated measurements at a single low-frequency (110 MHz) yielded essentially identical OP errors as a broadband frequency sweep (35 evenly spaced frequencies between 50 and 253 MHz) for these noise models. The inclusion of modulation frequencies >300  MHz diminished overall performance for one of the instruments. Additionally, we developed a LUT inversion algorithm capable of increasing inversion speeds by up to 6×, with 1000  inversions/s and ∼1% error when a single modulation frequency was used.

**Conclusion:** These results suggest that simpler single-frequency systems are likely sufficient for many applications and pave the way for a new generation of simpler digital FD-DOS systems capable of rapid, large-volume measurements with real-time feedback.

## 1 Introduction

Diffuse optical spectroscopy (DOS) is an optical technique that utilizes multiply scattered light to measure biological tissues. Frequency domain DOS (FD-DOS) is a variant, in which the light source is temporally modulated at radio frequencies (e.g., 50 to 1000 MHz). By measuring the changes in amplitude and phase of the optical signal after it passes through tissue, the absorption ($\mu_{a}$) and reduced scattering ($\mu_{s}^{\prime }$) coefficients, collectively termed optical properties (OPs), can be determined. Reduced scattering coefficients provide information on tissue microstructure,^1^ whereas $\mu_{a}$ can be used to estimate concentrations of tissue chromophores such as oxy-hemoglobin, deoxy-hemoglobin, lipid, and water. Hemodynamic information can give insight into tissue metabolism which has proven useful in tracking breast cancer response to treatment,^2^^,^^3^ has revealed new information about functional activation in the brain,^4^^–^^6^ and can be used to monitor metabolic activity in both muscles^7^ and joints.^8^

Research groups have taken different strategies for the selection of modulation frequencies for FD-DOS based on balancing the need to accumulate measurable phase shift, which is easier at higher frequencies, with the need to maintain measurable modulation amplitude, which is easier at lower frequencies. Mathematically, only a single modulation frequency and single source–detector separation is required to disentangle $\mu_{a}$ from $\mu_{s}^{\prime }$,^9^ and many instruments use a single modulation frequency, often with heterodyne detection to generate a beat frequency so lower-speed electronics can be used.^10^ In the early 1990s, a network analyzer-based FD-DOS measurement technique was developed that has since found frequent use for some clinical applications.^11^ The network analyzer allowed the measurement of frequency sweeps with hundreds of modulation frequencies over a wide bandwidth (e.g., 50 MHz to 1 GHz) at the expense of acquisition speed. In between these two extremes, other groups have used two,^12^ or several dozen modulation frequencies to measure $\mu_{a}$ and $\mu_{s}^{\prime }$.^13^

Previous work has investigated the theoretical limits of spatial resolution and sensitivity of FD-DOS measurements for a given single modulation frequency,^14^ and there have been investigations to determine an optimal modulation frequency for tomographic reconstruction assuming a shot-noise limited system.^15^^–^^17^ These prior works agree that, if a shot-noise limited system is available, an optimal modulation frequency exists between 300 and 800 MHz depending on the OPs of the sample and the source–detector separation employed. One of these works^15^ analyzed Fourier transformed time-domain data which may have higher contrast-to-noise ratio than devices operating in the frequency domain. Unfortunately, other important practical considerations, such as instrument bandwidth (i.e., the degradation of a system’s ability to generate and detect signals as frequency increases) and other sources of noise, were not taken into account in these analyses. Since real-world systems are highly constrained by instrument bandwidth, among other considerations, a practical analysis of modulation frequency choice and bandwidth remains elusive.

There has been less work done on the advantages of using more than a single modulation frequency. One group made use of an instrument-specific noise model to show that use multiple modulation frequencies for FD diffuse optical tomography can increase reconstruction accuracy compared with single-frequency data.^12^^,^^18^ Due to instrumentation limitations, they restricted their investigation to frequencies below 400 MHz.

In this work, we investigate how the choice and use of up to 450 separate modulation frequencies in the range of 50 to 500 MHz impacts the accuracy of optical property (OP) extractions for real-world FD-DOS systems when only a single source–detector separation is considered. The analysis takes into account both model-based errors as well as instrument characteristics. In the following sections, we first investigate how inverse model parameters affect OP errors when using noise-free simulations. After identifying optimal model parameters, we then use simulated data, both with and without added instrument noise generated from experimentally derived noise models to compare the impact of using different sets of modulation frequencies on OP recovery error for homogeneous media. Additionally, we also describe the development of a rapid multi-frequency look-up table (LUT) that dramatically increases inversion speeds compared to the standard iterative method. Finally, we discuss our results in the context of prior work and describe how other research groups can generate their own instrument specific noise models to conduct similar analyses. These results are highly relevant for researchers developing their own FD-DOS systems, and/or trying to decide how many and which modulation frequencies to use in their research.

## 2 Methods

### 2.1 Optical Property Estimation

**Fig. 1:**
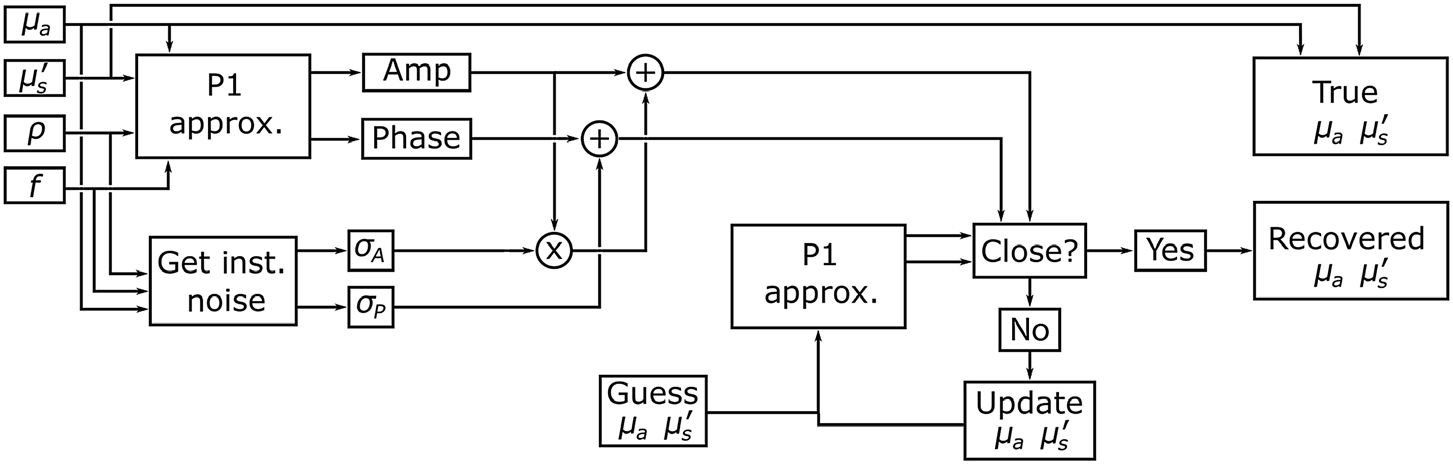
Flowchart for measuring OP recovery error. $\mu_{a}$ is the absorption coefficient, $\mu_{s}^{\prime }$ is the scattering coefficient, ρ is the source–detector separation, f is the modulation frequency. $\sigma_{A}$ is the standard deviation of amplitude determined from repeat measurements using a particular instrument. $\sigma_{P}$ is the standard deviation of phase measurements. The flowchart depicts how simulated data are generated and used to assess OP extraction error.

Light transport in tissue is well described by the radiative transport equation (RTE). For highly scattering media, the P1 approximation to the RTE can be used, for which there are analytical solutions for many common geometries.^19^ In this study, we use the P1 approximation to the RTE for a semi-infinite geometry to generate the amplitude and phase response for a range of OPs, source–detector separations, and modulation frequencies.^20^ Details on the solution to the P1 approximation have been previously published,^20^ and for our specific implementation see the “Code and data availability” section. This model assumes that the underlying sample is homogeneous, so in complex biological tissue represents the bulk averaged OPs. In this study, we use an iterative inversion model to estimate OPs from experimental and simulated data and show how changing the parameters of this model can have a large effect on OP error. Later, we compare a LUT inversion model, that makes use of the same P1 approximation, with the iterative model in both speed and accuracy. A flowchart of the method for generating simulated data for a single source–detector separation and recovering OPs from that data is shown in Fig. 1. We use the P1 approximation to simulate the amplitude and phase response given randomly selected values of $\mu_{a}$ and $\mu_{s}^{\prime }$. Noise is then added to this response based on noise models generated for each of the two FD-DOS instruments. In order to recover $\mu_{a}$ and $\mu_{s}^{\prime }$ from the simulated data, we use the Levenberg–Marquardt algorithm (LMA) to determine the OPs that minimize the squared error of the complex input and output.^21^ The LMA has been used in previous FD-DOS studies including our own.^20^ All the analysis presented here were performed using a set of 10,000 pairs of OPs that were randomly selected from a uniform distribution such that $\mu_{s}^{\prime }>10\mu_{a}$ to stay in the range where the P1 approximation is valid. The range of $\mu_{a}$ investigated was 0.001 to $0.05\;{\mathrm{mm}}^{-1}$ and the range of $\mu_{s}^{\prime }$ was 0.1 to $3\;{\mathrm{mm}}^{-1}$. This range of OPs covered many biological tissues in the near-infrared, including breast, brain, and muscle.^22^ The recovered OPs were compared with the true OPs by measuring their normalized distance according to Eq. (1), where $\mu_{\mathrm{at}}$ is the true absorption coefficient, $\mu_{\mathrm{ar}}$ is the recovered absorption coefficient, $\mu_{\mathrm{st}}^{\prime }$ is the true reduced scattering coefficient, and $\mu_{\mathrm{sr}}^{\prime }$ is the recovered reduced scattering coefficient. Equation (1) does not allow the errors in $\mu_{a}$ to be separated from errors in $\mu_{s}^{\prime }$ and was chosen to simplify visualization. We found that errors in $\mu_{a}$ and $\mu_{s}^{\prime }$ were highly correlated (Pearson coefficient of ∼0.6), and plots of $\mu_{a}$ and $\mu_{s}^{\prime }$ error were quite similar (see Supplemental Material):

$$d=\sqrt{{\left(\frac{\mu_{\mathrm{ar}}-\mu_{\mathrm{at}}}{\mu_{\mathrm{at}}}\right)}^{2}+{\left(\frac{\mu_{sr}^{\prime }-\mu_{\mathrm{st}}^{\prime }}{\mu_{\mathrm{st}}^{\prime }}\right)}^{2}}.$$ (1)

Both the forward and inverse models were implemented in MATLAB 2019a (The Mathworks, Natick, MA). The LMA algorithm can converge on either local or global minima. One way to increase the likelihood of converging onto the global minimum is to repeat the fitting algorithm with a different initial guess. We used six starting locations that spanned the OP space to increase the probability of finding the true minimum and selected the final result as the OP pair with the lowest residual error. In addition to the starting location, there are a number of additional options that are used to control the behavior of the LMA algorithm. We investigated a variety of stopping conditions before settling on an optimum set that yielded virtually zero error across the entire OP space for all modulation frequency conditions investigated under noise-free conditions. The default fit options were: $\mathrm{TolFun}=1\times 10^{-10}$, $\mathrm{TolX}=1\times 10^{-6}$, MaxIter=200, $\text{OptimalityTolerance}=1\times 10^{-6}$, $\text{StepTolerance}=1\times 10^{-6}$, whereas the optimized options were $\mathrm{TolFun}=1\times 10^{-35}$, $\mathrm{TolX}=1\times 10^{-20}$, $\mathrm{MaxIter}=1\times 10^{5}$, $\text{OptimalityTolerance}=1\times 10^{-10}$, and $\text{StepTolerance}=1\times 10^{-20}$. These functions adjust the stopping conditions of the LMA. TolFun is the minimum change in the value of the squared 2-norm of the difference between the experimental and simulated data before iterations stop. TolX and StepTolerance are the minimum change in $\mu_{a}$ and $\mu_{s}^{\prime }$ that are allowed before iterations stop. MaxIter is the maximum number of iterations that will be performed before the algorithm stops. OptimalityTolerance is a MATLAB-specific measure of how close the OP pair is to optimal. The default options have been used extensively in the previous work.^13^^,^^23^^–^^25^ We also implemented an additional check that excluded reconstructed OP pairs that did not meet the $\mu_{s}^{\prime }>10\mu_{a}$ criteria. This check was performed because the LMA algorithm would occasionally converge in the region where $\mu_{a}$ and $\mu_{s}^{\prime }$ were on the same order of magnitude. In the case of simulated data, we know that none of the true OPs fell outside of the $\mu_{s}^{\prime }>10\mu_{a}$ boundary, which might not always be the case in biological tissue.

The optimized parameters resulted in slower inversion rates (∼5  Hz) compared to the default parameters (∼50  Hz) on a desktop PC (Intel i9-9900). In order to increase the speed of OP calculation, we developed a multi-frequency LUT method. In this method, we used the P1 approximation to the RTE to precompute the complex-valued response for a dense grid of $\mu_{a}$ and $\mu_{s}^{\prime }$ pairs for all necessary modulation frequencies. The results were stored in two, 3-dimensional LUTs, one for the real part of the response and the other for the imaginary part of the response. In these LUTs, the dimensions represented $\mu_{a}$ values, $\mu_{s}^{\prime }$ values, and modulation frequencies. When experimental data were collected, or simulated data generated, the squared difference between the real and imaginary parts of the data and each element of the LUT were calculated for each modulation frequency. The results were then summed over all relevant modulation frequencies to obtain a single “goodness-of-fit” statistic ($\chi^{2}$) for each OP pair [Eq. (2)]. The recovered OP value was selected as the $\mu_{a}$ and $\mu_{s}^{\prime }$ combination at which the $\chi^{2}$ statistic was minimized:

$$\chi^{2}=\underset{f=f_{0}+mf_{s}}{\sum }\sqrt{{\left(Re(r_{f})-Re(z(f,\mu_{ai},\mu_{sj}^{\prime }))\right)}^{2}+{\left(Im(r_{f})-Im(z(f,\mu_{ai},\mu_{sj}^{\prime }))\right)}^{2}.}$$ (2)

In Eq. (2), m=0,1,2…,n, where n is the number of modulation frequencies in the measurement, $f_{0}$ is the starting frequency, $f_{s}$ is the frequency step, $r_{f}$ is the experimental data at frequency f, $z(f,\mu_{ai},\mu_{sj}^{\prime })$ is the complex result of the forward model at frequency f for the OP pair ($\mu_{ai}$, $\mu_{sj}^{\prime }$) and represents a single element of the LUT.

This method requires that $\chi^{2}$ be calculated for every OP pair, which implies a trade-off between speed and accuracy as the number of precalculated OPs is changed. We found that a 500×500 grid of $\mu_{a}$ and $\mu_{s}^{\prime }$ values spanning the OP space described above yielded sufficiently fast inversions to process data at video rates (30 Hz) with reasonable accuracy.

For both the iterative and LUT approaches, experimental data must be calibrated. One method for doing this is to measure a phantom with known optical properties to calculate the instrument response function (IRF). Once the IRF is removed from the measurements, the calibrated data are appropriately scaled to the forward model used for calibration.

### 2.2 Experimental Setup

The performance of each of the three inversion models in the best case scenario was determined using noise-free simulations. In order to assess their performance in real-world conditions, experimental noise models were developed for two broadband frequency-domain instruments. In the following section, we describe the two instruments and discuss how the noise models were constructed.

The first FD-DOS instrument investigated used a commercial network analyzer (Agilent Technologies, Palo Alto, California) to measure amplitude and phase and has been described in detail elsewhere.^20^ Briefly, six laser diodes (658, 690, 785, 808, 830, and 850 nm) were sequentially supplied with an radio frequency (RF) modulation current that ranged between 50 and 500 MHz. The modulated light was delivered to the sample using an optical fiber bundle. A 1-mm active area avalanche photodiode (APD) was positioned 28 mm from the source fiber in direct contact with the sample to detect remitted light. The network analyzer computed the amplitude and phase of the analog signal delivered from the APD detection module. A similar instrument setup has been previously used for clinical studies,^11^^,^^24^^,^^26^ where ∼400 modulation frequencies were used for each measurement. The same laser and source fibers were used for both systems, so the total laser power should be identical between the two.

The second instrument we investigated was a custom, all-digital, FD-DOS system previously developed in our laboratory.^25^ Instead of a network analyzer, this system used six direct digital synthesis boards to simultaneously supply modulation signals to each of the laser diodes. All six lasers illuminated the tissue simultaneously, and the signal from each laser was demultiplexed using signal processing conducted in the frequency domain. The light was delivered to the tissue using an optical fiber bundle of 400  μm fibers, and a second 2.5-mm-diameter fiber, placed at 10, 20, or 30 mm from the source, was used to deliver remitted light to a custom APD module with a 3-mm active area APD. The electrical signal from the APD was digitized by a high-speed (250  megasample/s) analog to digital converter for processing. Amplitude and phase information are preserved even after aliasing allowing this system to analyze signals above the Nyquist frequency.^25^ Frequencies that alias to 0 Hz (i.e., multiples of 125 MHz) must be avoided as, in this special case, information about phase and amplitude is lost. The combination of wavelength multiplexing and rapid frequency switching enabled by the digital electronics allowed data to be collected at up to 100 Hz. The all-digital system also allows for a highly flexible frequency selection paradigm, in which any combination of a nearly unlimited number of frequencies between 1 and 500 MHz could be used.

Experimental noise metrics for both systems were derived from repeated measurements collected from two tissue simulating optical phantoms, one with a relatively high absorption ($\mu_{\mathrm{aH}}=0.02\;{\mathrm{mm}}^{-1}$, $\mu_{s}^{\prime }=0.53\;{\mathrm{mm}}^{-1}$ at 850 nm) and one with a relatively low absorption ($\mu_{\mathrm{aL}}=0.003\;{\mathrm{mm}}^{-1}$, $\mu_{s}^{\prime }=0.81\;{\mathrm{mm}}^{-1}$ at 850 nm). For the all-digital system, a single measurement consisted of 65,536 samples from the ADC at each modulation frequency. These time varying data, collected over 260  μs, were used to calculate a single amplitude and phase value for each frequency. For the network analyzer system, a single measurement consisted of an amplitude and phase measurement at each frequency calculated by the network analyzer. The optical fibers were not repositioned between measurements. Data were collected over a frequency range of 50 to 500 MHz for both systems and the distribution of measured data was recorded. Examination of quantile–quantile plots of amplitude and phase from the repeated measurements was linear indicating that they could be described by a normal distribution.

Examination of the experimental data revealed that the standard deviation of the amplitude and phase depended on the modulation frequency, absorption coefficient, wavelength, and source–detector separation with the noise increasing as signal was reduced. This trend is opposite what one would expect in a shot-noise dominated system. These factors are those that most directly impact the amount of light returning from the tissue which appears to be closely related to the measured noise. Standard deviation values for arbitrary frequencies were estimated by smoothing the standard deviation versus frequency curve with a moving average filter and interpolating values between points of the smoothed curve using a shape preserving piecewise cubic scheme.

To determine the effect of optical properties on the noise level, we measured 17 phantoms with $\mu_{a}$ ranging from 0.003 to $0.03\;{\mathrm{mm}}^{-1}$ and $\mu_{s}^{\prime }$ between 0.4 and $1.4\;{\mathrm{mm}}^{-1}$. We found that both amplitude and phase noise levels were strongly correlated with $\mu_{a}$ (Pearson correlation coefficient of 0.8), while it was not correlated with scattering (correlation coefficient of 0.2). We found that noise level was approximately linearly related to $\mu_{a}$ ($R^{2}$ of 0.75). In these studies, only a single wavelength (850 nm) and source–detector separation (30 mm for the digital system and 28 mm for the network analyzer system) was used and there was no attempt to simulate data at different wavelengths or source–detector separations.

The final noise level in either amplitude or phase (σ) for an arbitrary value of $\mu_{a}$ was determined by fitting a line to the measured $\mu_{a}$ versus noise data at each frequency (f) for the low-absorbing ($\mu_{aL}$) and high-absorbing ($\mu_{aH}$) phantom and using the value of the simulated $\mu_{a}$ to scale the noise level based on those fits:

$$\sigma (f,\mu_{a})=\frac{\sigma (f,\mu_{aH})-\sigma (f,\mu_{aL})}{\mu_{aH}-\mu_{aL}}(\mu_{a}-\mu_{aL})+\sigma (f,\mu_{aL}).$$ (3)

### 2.3 Data Analysis

Six different modulation frequency scenarios were investigated using noise-free and noisy data. Three of the six scenarios used a single modulation frequency: 70 MHz was chosen based on an instrument described in Ref. 4, 110 MHz was chosen as this is the frequency used by instruments produced by ISS Inc. (Champaign, IL, USA), and 500 MHz was chosen to help discern the effect of using a relatively high frequency as suggested by previous literature.^15^ We also investigated three additional scenarios, in which more than one modulation frequency was used. These included a scenario with two widely spaced modulation frequencies (50 and 500 MHz): a scenario with 30 modulation frequencies from 50 to 253 MHz in steps of 7 MHz, which is a combination of frequencies used in our previous work,^13^ and scenarios with 450 frequencies between 50 and 500 MHz in steps of 1 MHz, which has been widely used for prior broadband FD-DOS studies of breast cancer therapy monitoring.^3^

For each set of modulation frequencies for the scenarios described above, 10,000 OP inversions were conducted two times. In the first run, the same overall amount of data was used for each OP inversion regardless of the modulation frequency set. We refer to this as the “equal data” condition. This procedure was employed to ensure that scenarios with more than one modulation frequency would not have an advantage because they included more data per measurement. To accomplish this, 450 data points were generated for each pair of OPs and for each of the six scenarios described above. For the five scenarios with fewer than 450 modulation frequencies, measurements were simulated multiple times, each time with noise levels drawn randomly from the distributions described in Sec. 2.2. This was done to mimic a situation, in which the same measurement was repeated multiple times. The amplitude and phase data from each of these simulations were averaged prior to applying the inverse model. For example, for single modulation frequency scenarios, measurements at that modulation frequency were simulated 450 times with random noise added to each, the amplitudes and phases from each simulation were averaged. In experimental measurements, each modulation frequency is collected independently with identical integration time and the same amount of light energy incident on the sample which ensures that no matter how many frequencies were used, comparisons between conditions can be made. OPs were estimated as described above using six starting locations for the LMA. Of the six candidate OP pairs generated from this method, the pair with the lowest residual error was saved. In the second run, each measurement was simulated a single time no matter how many modulation frequencies were employed. We refer to this as the “unequal data” condition because the number of simulated data points varied depending on the number of modulation frequencies used.

For each of the modulation frequency scenarios, simulated data were generated using noise-free data, and using experimental noise models for the all-digital system at three different source–detector separations (10, 20, and 30 mm), and the network analyzer system at 28 mm. OP extraction accuracy was compared between these scenarios for the different inversion models.

## 3 Results

### 3.1 Inversion Models

**Fig. 2:**
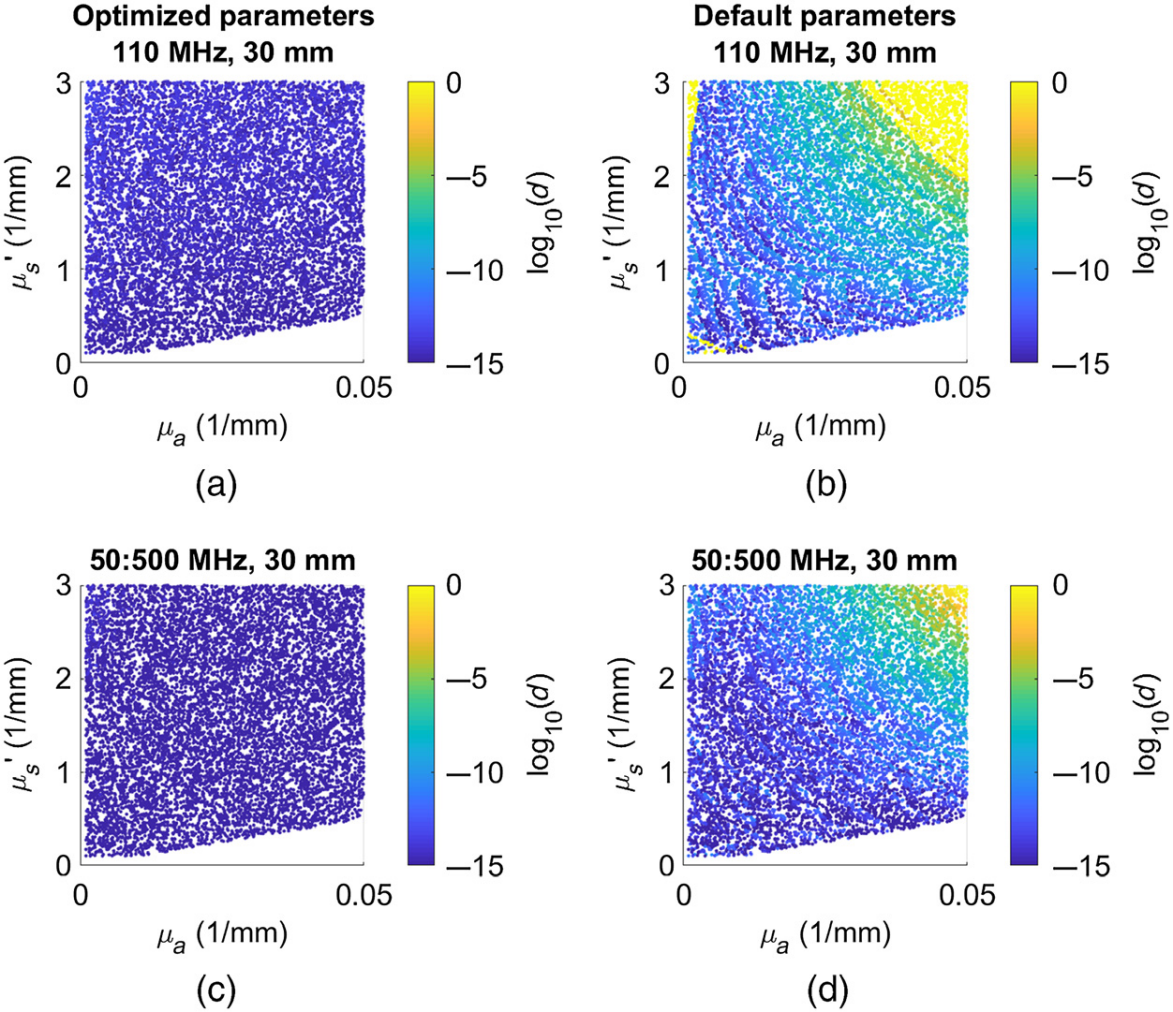
Error landscape plots for noise-free simulations at 110 MHz and 30 mm source–detector separation using (a) optimized fit parameters and (b) default fit parameters. Multi-frequency error landscape plots from noise-free simulations using 451 modulation frequencies from 50 to 500 MHz in steps of 1 MHz using the (c) optimized fit parameters and (d) default fit parameters. The blue color indicates near perfect OP extraction, whereas the yellow region shows areas of large errors. Error values are labeled d as defined in Eq. (1) and map to percent error with $\log_{10}(d)=0.15$ equivalent to 100% error in both $\mu_{a}$ and $\mu_{s}^{\prime }$. Error landscapes for the default parameters plotted as percent error for $\mu_{a}$ and $\mu_{s}^{\prime }$ separately are shown in Fig. S1 in the Supplemental Material.

Figure 2 shows the importance of selecting the optimized LMA fit options. To visualize the quality of OP extractions, all 10,000 OP pairs are plotted as points on a single graph with $\mu_{a}$ on the abscissa and $\mu_{s}^{\prime }$ on the ordinate. Each point is assigned a color based on the normalized distance between the true OP pair used to generate the synthesized data and the OP estimate recovered by the inverse model [Eq. (1)]. Using the default options, the error for some OP combinations was over 70%, as indicated by values were $\log_{10}$ (error) > 0 on the plot. These results are representative for all modulation frequencies at 30 mm. As the source–detector separation decreases, the error using the default options reduced steadily. Surprisingly, at 10-mm source–detector separation, the optimized options for single-frequency conditions have a small region of higher error when both $\mu_{a}$ and $\mu_{s}^{\prime }$ are low. Overall, the measured errors in Fig. 2 using the default parameters are low (less than about 10%). However, for noise-free simulations, the error should be on the order of the precision of floating point numbers ($∼10^{-16}$). The presence of errors up to 10% from just the inversion model has the potential to overshadow instrument-dependent errors, potentially complicating the assessment of the performance of the two instruments using different modulation frequency scenarios. These error landscapes are shown as percent error for $\mu_{a}$ and $\mu_{s}^{\prime }$ separately in Fig. S1 in the Supplemental Material.

**Fig. 3:**
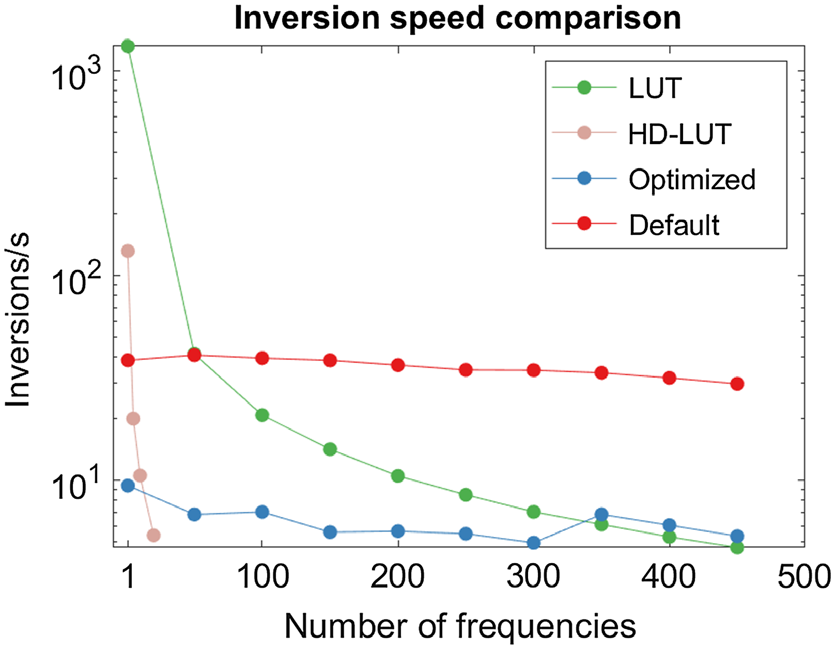
Inversion speed for the different inversion methods employed presented on a semilog scale. The iterative methods are insensitive to number of frequencies while the 500×500 point LUT and 2000×2000 point HD-LUT speed decrease linearly as frequencies are added.

The optimized LMA model is significantly slower than the default LMA model due to the increase in the number of iterations allowed as well as the tighter tolerances imposed. We measured the inversion speed for the standard LMA algorithm, the optimized LMA algorithm, and the LUT approach in MATLAB (Fig. 3). We found that the optimized parameters resulted in a 6× slower inversion speed (6 versus 36 Hz on average) and that the speed of the iterative models was not affected by the number of frequencies used. The LUT approach speed decreased linearly as the number of modulation frequencies was increased due to the fact that additional frequencies linearly increased the size of the LUT. However, when only a single frequency was used, the LUT approach was capable of performing over 1000 inversions per second, much faster than either iterative method. As the number of frequencies was increased, the speed advantage was also reduced, matching the default LMA algorithm at 50 frequencies, and the optimized algorithm at 350 frequencies. Use of a high-density LUT (HD-LUT) using a 2000×2000 point grid of $\mu_{a}$ and $\mu_{s}^{\prime }$ values resulted in a faster degradation of inversion speed as additional frequencies are added. Although such analysis is beyond the scope of the current work, we anticipate that significantly higher performance could be achieved using C/C++ or other compiled languages. We are unsure exactly why the iterative method execution time is insensitive to the number of frequencies used. One possibility is that the calculation of amplitude and phase is vectorized so the difference between calculating the response to one frequency versus 400 frequencies in MATLAB is negligible. Conversely, the LUT method uses less efficient computation in its search for the closest OP pair.

### 3.2 Instrument Noise

**Fig. 4:**
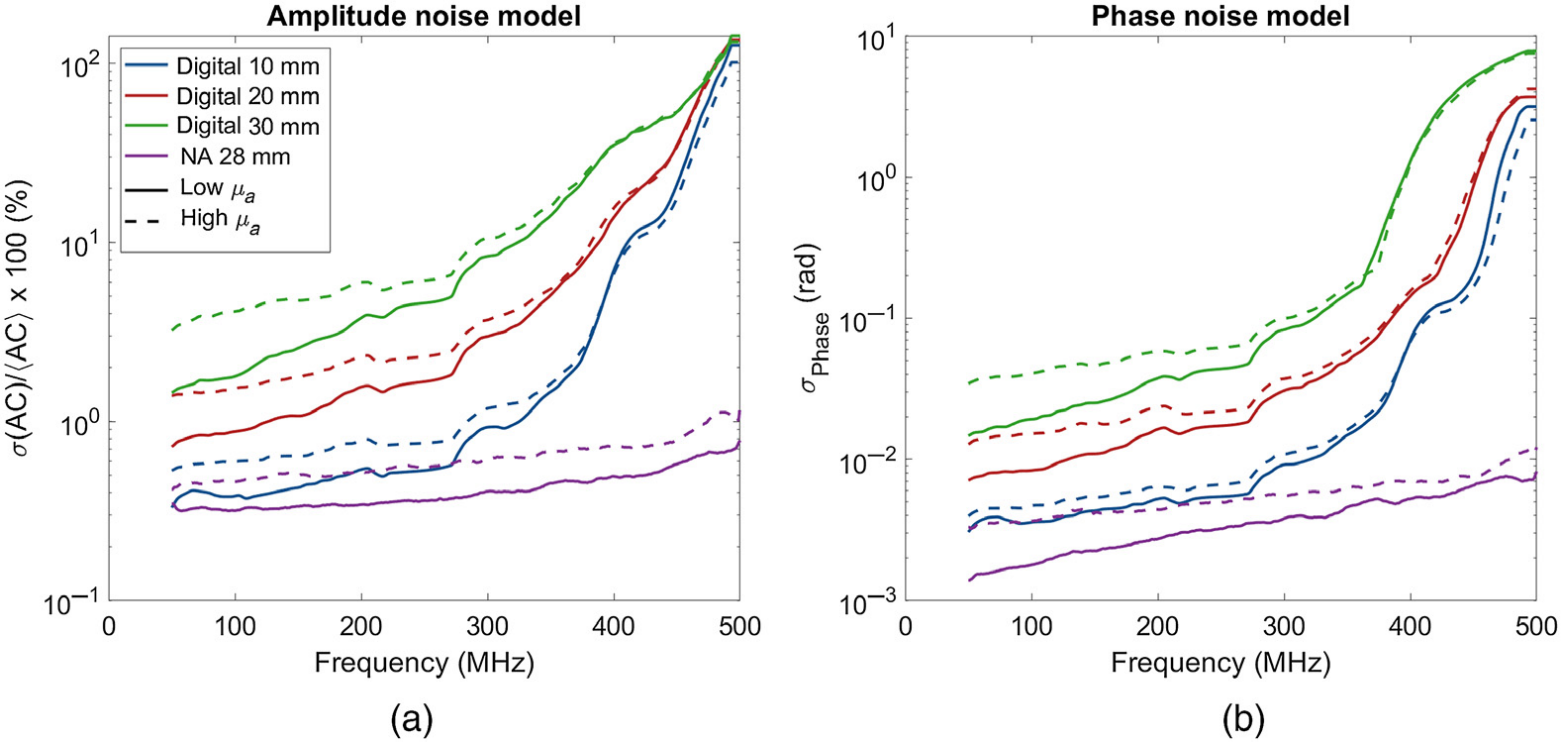
(a) Amplitude and (b) phase noise models for the two different instruments investigated presented on a semilogarithmic scale. The digital curves were smoothed with a 5-point moving average filter, and the NA curve was filtered with a 15-point moving average filter. Dashed lines indicate measurements on the high-absorption sample ($\mu_{a}=0.03$) and solid lines indicate the low-absorption sample ($\mu_{a}=0.003$).

To determine the instrument-dependent noise, we first measured the standard deviation of raw amplitude and phase data over repeated measurements. The use of repeated measurements to estimate the noise characteristics was necessary because the all-digital system was incapable of storing more than 65,536 points due to memory limitations. Similarly, the network analyzer-based system was programmed to output amplitude and phase calculations periodically. Figure 4 shows the normalized standard deviation, also known as the noise to signal ratio (σ(AC)/⟨AC⟩) of these measurements as a function of modulation frequency for the high-speed digital system for both the low $\mu_{a}$ phantom (solid lines) and high $\mu_{a}$ phantom (dashed lines). The network analyzer system had the lowest noise across all modulation frequencies and was relatively flat in amplitude as frequency increased. The digital system, on the other hand, showed a sharp increase in amplitude noise above ∼300  MHz. This was likely due to a combination of the lower radio frequency power supplied by the digital synthesis boards at higher modulation frequencies, the decreased modulation amplitude of the lasers at higher frequencies, and the lower detector sensitivity at higher frequencies. The digital instrument had higher phase noise than the network analyzer system and showed a sharp increase above 350 MHz. The network analyzer system had lower phase noise overall that increased gradually with frequency.

**Fig. 5:**
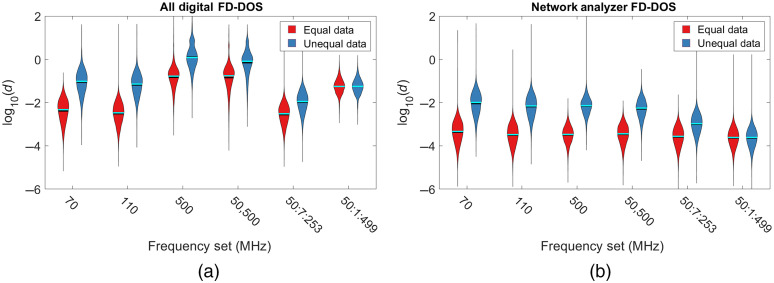
Violin plots for the (a) digital FD-DOS system at an SD separation of 30 mm and (b) network analyzer system at an SD separation of 28 mm showing the distribution of OP inversion errors [labeled d as defined in Eq. (1)] using equal numbers of data points for each condition (red), or only a single simulated measurement (blue). For the digital system, repeated measurements at 110 MHz and frequencies below 300 MHz resulted in the lowest errors, whereas the network analyzer system was most accurate when all frequencies were used. For single measurements using the digital system, additional frequencies lowered the error so long as high-noise frequencies above 300 MHz were not used. For all plots, the optimized fitting parameters were used.

Using the optimized inversion model, we can be confident that errors in OP extraction are overwhelmingly due to system noise. Figure 5 shows the distribution of the OP extraction errors when using the optimized parameters for the digital instrument (a) and the network analyzer (b) using violin plots. Each violin shows the error distribution of the 10,000 OP pairs encoded in the width of the bar. The black lines represent the mean of the distribution and the cyan bars represent the median. The broad distribution of error is driven by a few outliers at the extremes of the distribution. Broadly, low values for $\mu_{a}$ and $\mu_{s}^{\prime }$ resulted in smaller extraction errors. There are two distributions for each modulation frequency set that correspond to the two different methods of data simulation. The red equal data bars were constructed using the scheme where every modulation set used the same number of data points. The blue unequal data bars were constructed using the simulation of only a single measurement which is similar to how data have been collected in the previous studies.^2^ We caution readers that the unequal data condition compares acquisitions with different integration times and different amounts of data, and this is included given that it reflects current practice in the field. We found that for the equal data case, repeated measurements at 110 MHz yielded essentially identical errors to the low-frequency broadband sweep (i.e., 50:7:253 MHz). The digital system had an error of $\log_{10}(d)=-2.5\pm 0.5$ at 110 MHz versus $\log_{10}(d)=-2.6\pm 0.5$ using 50:7:253. The network analyzer system had an error of $\log_{10}(d)=-3.5\pm 0.5$ at 110 MHz versus $\log_{10}(d)=-3.6\pm 0.5$ using 50:7:253. When only a single measurement was simulated, there was a modest benefit from using multiple frequencies especially if high-noise frequencies above 300 MHz were avoided as in the 50:7:253 MHz scenario [$\log_{10}(d)=-2.0\pm 0.5$ for the digital system]. Since the increase in noise as a function of frequency is less pronounced for the network analyzer system, when only a single measurement was used, the sweep containing the most frequencies had the best accuracy [$\log_{10}(d)=-3.6\pm 0.4$]. These results indicate that while additional data are valuable for accurately extracting $\mu_{a}$ and $\mu_{s}^{\prime }$, it may be advantageous to gather that additional data from repeated measurements at a single modulation frequency rather than using a broad frequency sweep, especially when frequencies with high-noise levels, such as those >300  MHz for the digital system, were included. The advantage of using multiple measurements at a single frequency is beyond what would be expected from simply using more light energy or more integration time. If additional light energy was the driving factor, then more frequencies would result in lower error which we do not find for the digital system (Fig. 5). The network analyzer system does show a reduction in error as more frequencies are added which emphasizes the fact that instrument-specific noise models are needed to appropriately select optimal modulation frequencies.

**Fig. 6:**
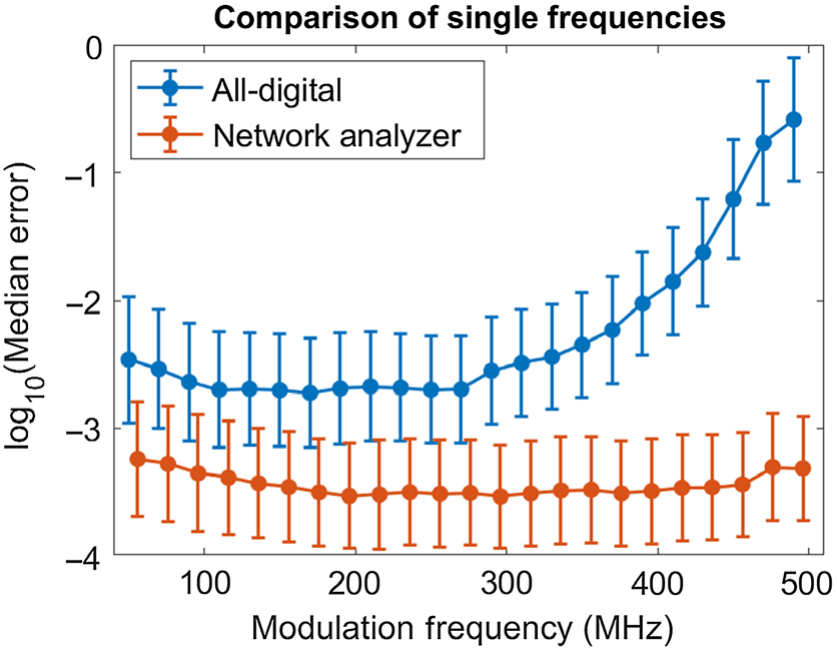
Comparison of different single modulation frequencies between the two instruments. 290 MHz is the optimal frequency for the network analyzer system, whereas 170 MHz is the optimal frequency for the digital system. However, there is a broadband where the error is basically static between 100 and 250 MHz. Error bars represent ± one standard deviation of $\log_{10}$ transformed error. Here “median error” refers to the median value of error metric d defined in Eq. (1). Two extreme outliers were removed from the all-digital system at 410 MHz. Network analyzer points have been shifted by 6 MHz for clarity.

Figure 6 shows a comparison of different single modulation frequencies and the resulting error. It demonstrates that there is a range of frequencies that have very similar error characteristics. For the digital system, this band is between 100 and 250 MHz; whereas for the network analyzer system, the band is between 150 and 300 MHz.

**Fig. 7:**
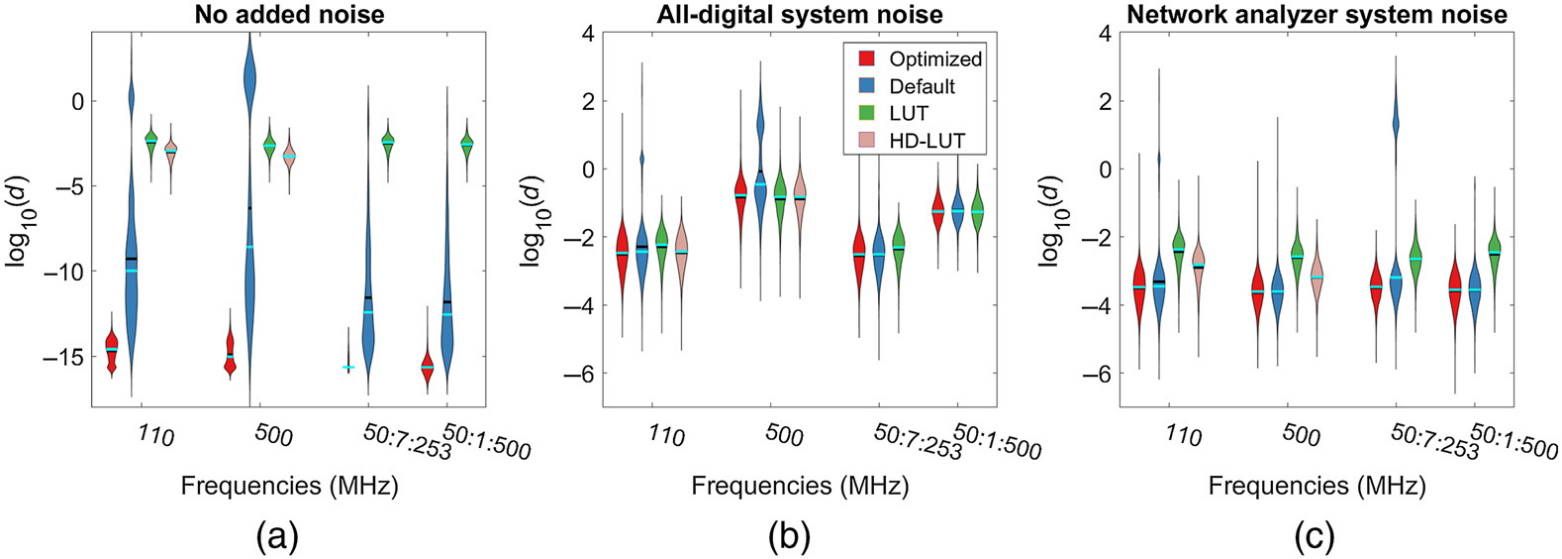
Reconstruction accuracy [with error labeled d as defined in Eq. (1)] comparisons between different inversion methods with different noise models. (a) The noise-free simulations where the optimized iterative method performs better. (b) The performance of each algorithm with added noise similar to the all-digital system. (c) is identical to (b) except noise similar to the network analyzer system was used. The LUT method (green) used a 500×500 point table shows slightly higher error on average than the iterative method. For single frequencies, we also show the error when an HD-LUT consisting of 2000×2000 points was used. For a single modulation frequency, the inversion speed of the HD-LUT is ∼5× faster than the default iterative method.

Finally, we examined the errors associated with all three inverse models (Fig. 7). In this figure, each inversion method is assigned a color and the distribution of error is shown using violin plots. We found that in the single-frequency noise-free case [Fig. 7(a)], the optimized iterative method is able to estimate OPs with less error than the default method indicated by the fact that the maximum value of the error metric was $∼10^{-13}$ for the optimized method while it was nearly 2 for the default method. When noise was added, the optimized method still results in lower error due to a cluster of high-error points that are found when both $\mu_{a}$ and $\mu_{s}^{\prime }$ are high. Outside of those regions, the optimized and default inversion methods show similar performance [Figs. 7(b) and 7(c)]. With multiple modulation frequencies, the optimized iterative method only outperformed the default in the noise-free case. We also found that the LUT method has higher errors that are relatively consistent across all added noise conditions. Figure 7 can be used to help inform which inversion model should be used based on the needs of a particular application. If the highest accuracy is required, the optimized iterative method should be used. If the fastest speed is needed, the LUT method may be the best choice. The default iterative method can provide good performance under most conditions, but is susceptible to very large errors especially on samples with both high $\mu_{a}$ and $\mu_{s}^{\prime }$.

**Fig. 8:**
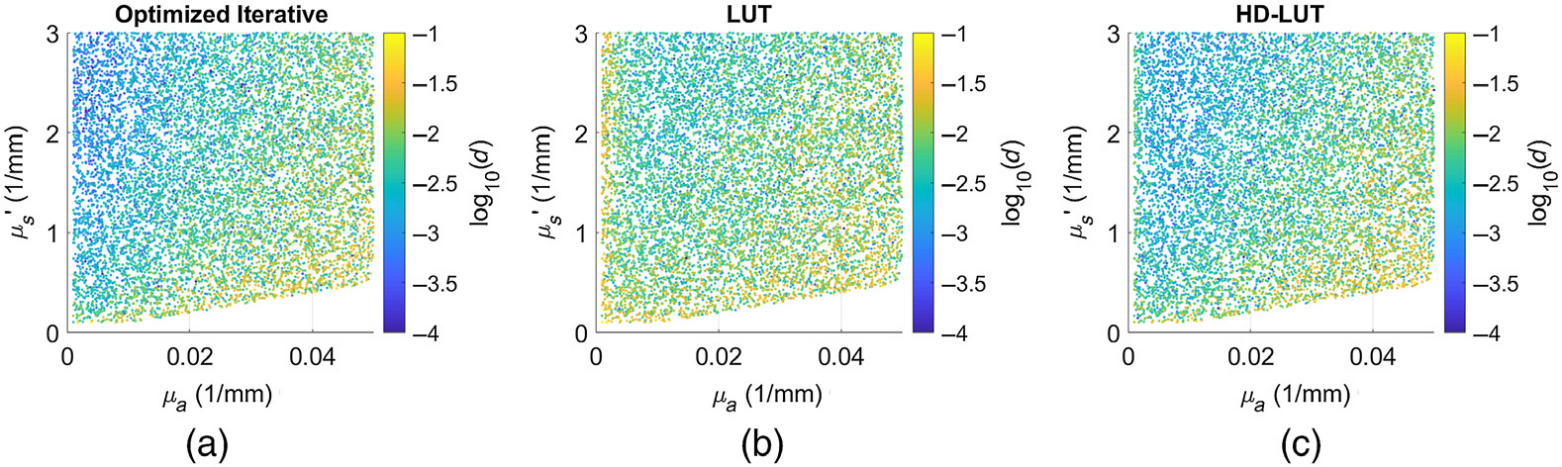
Error landscapes for the digital system noise model using (a) the optimized iterative method; (b) the 500×500 point LUT; and (c) the 2000×2000 point LUT at 100 MHz. In the plots, the effect of $\mu_{a}$ can be observed as an increasing amount of error [labeled d as defined in Eq. (1)] moving from left to right in each figure. The HD-LUT shows lower error than the LUT and is comparable to the iterative method. These error landscapes plotted as percent error for $\mu_{a}$ and $\mu_{s}^{\prime }$ separately are shown in Fig. S2 in the Supplemental Material.

Finally, we illustrate the performance of the LUT and HD-LUT methods in Fig. 8. In this figure, we show the iterative, LUT, and HD-LUT methods using the digital system’s noise model. The effect of $\mu_{a}$ on simulated noise can be visualized by a generally increasing amount of error moving from left to right. The LUT method has a region of high error when $\mu_{a}$ is very low which vanishes when the higher density LUT is used. Overall, the HD-LUT has very similar error characteristics to the optimized iterative method and runs in approximately one fifth the time. The clustering of high-error regions in these plots is less distinct than in Fig. 2 due to the fact that these plots show the analysis of data with the addition of noise. The amount of noise added to any particular point is randomly determined which can lead to adjacent points having different levels of error. These error landscapes plotted as percent error for $\mu_{a}$ and $\mu_{s}^{\prime }$ separately are shown in Fig. S2 in the Supplemental Material.

## 4 Discussion

We have demonstrated that, for the instruments and light transport model tested, there is no advantage to using a broad modulation frequency sweep rather than repeated measurements at a single modulation frequency when trying to minimize OP extraction errors for FD-DOS measurements on homogeneous media. In fact, the use of noisy, high-frequency modulation data as part of a broadband measurement, can lead to reduced accuracy.

This work suggests a markedly different modulation choice strategies than previous studies. For the instruments investigated, we have found that repeated measurements at a single, relatively low modulation frequency, provides the most accurate OP reconstruction for 2D topographic imaging. Previous research that converted time-domain signals to the frequency-domain has demonstrated that frequencies above 500 MHz are preferable based on a theoretical model of instrument noise that assumes shot noise is the dominate noise source.^15^ We attribute this difference to our use of a more realistic noise model that takes into account other noise sources such as electronic noise, 1/f noise, and other sources of white noise, in addition to shot noise.

Though we can only draw firm conclusions about the two instruments investigated, we believe that this result is more broadly generalizable. The foundation of these results is that higher modulation frequencies have a lower signal-to-noise ratio (SNR) than lower frequencies. We believe that this trend will be true for any FD-DOS instrument because higher frequencies are more challenging to generate and detect than lower frequencies due to intrinsic and parasitic capacitance which results in a lower amplitude and reduced signal. In addition, high-frequency photon density waves attenuate more quickly than low-frequency photon density waves which would also lead to lower signal. The methods presented in this paper provide other researchers with a straightforward way to determine the optimal modulation frequency for their FD-DOS systems.

For researchers wishing to improve their OP extraction accuracy, we recommend that they take repeated measurements from an optical phantom. These measurements can be used to track the frequency-dependent noise characteristics and develop a noise model for their systems. The linearity of QQ-plots of the amplitudes and phases at each frequency can be examined to determine if the noise is Gaussian in nature. For non-Gaussian noise, researchers can develop custom probability distributions to draw from. These data can be used to produce a figure similar to Fig. 4 which they can use to make a first approximation of the optimal frequencies to use. The presence of a corner frequency where the noise starts to increase rapidly will define the maximum practical frequency of the system. It is not certain that the frequency with the lowest measured noise will be the optimal frequency, so we further recommend modeling a range of single frequencies to generate a plot similar to Fig. 6. This plot should provide the range of optimal frequencies to use. Repeated measurements at one of those frequencies or sweeps within that range should provide equivalent OP extraction accuracy.

These results have important implications for the design of future FD-DOS instruments. Broadband frequency sweeps, by necessity, require electronics and detectors with a wide frequency response which can allow additional 1/f, or white noise into the signal. With an instrument utilizing only a narrow band of frequencies, band pass filtering or lock-in amplification can be used to dramatically improve the SNR of the measurements. In preliminary experiments using a single modulation frequency, we found that the addition of an analog bandpass filter with a passband centered at 145 MHz placed immediately before digitization in the all-digital system increased SNR by about 3 dB by removing high-frequency noise that may alias down into the spectral band of interest. When only a single frequency is employed, it is possible to use lock-in amplification which has the potential to improve SNR by tens of decibels.^27^ In addition to hardware improvement, single-frequency acquisition enables the averaging of more measurements while maintaining the same acquisition speed. This additional averaging should lead to improved SNR by reducing the noise by a factor of $\sqrt{n}$, where n is the number of measurements that are averaged.

Finally, we note that the combination of the new LUT inversion method with repeated single modulation frequency measurements leads to potentially very high-speed FD-DOS acquisition and real-time processing. We have previously shown that our digital FD-DOS system can acquire single modulation frequency, six wavelengths measurements at speed >1  kHz.^25^ The LUT method presented here can perform OP inversions at a similar 1 kHz rate, although this rate would decrease when multiple wavelengths are processed simultaneously. Using an iterative method, video rate FD-DOS imaging would be impossible at the current time using MATLAB because the inversion rate is too slow (30 and 6 Hz for the default and optimized parameters, respectively), though hand optimized compiled code could improve this speed. Although most biological phenomena happen significantly more slowly than 1 kHz, high-speed measurements can be used to rapidly image large volumes of tissue.^28^ One problem identified in a recent multi-center clinical trial of FD-DOS was poor data quality necessitating the removal of some patients from the analysis.^2^ High-speed OP extractions could enable real-time visualization of tissue contrast which would allow the operator to ensure that high-quality data were being collected.

We note that there may still be a benefit to broadband frequency sweeps for diffuse optical tomography or multi-layered media which were not investigated here. More complex geometries may have non-unique solutions when only a single frequency is employed and might benefit from the use of more modulation frequencies. Similarly, other groups utilize multi-distance measurements^29^ which do not require calibration on a known sample to remove the IRF. Our study did not attempt to determine the effect of source modulation frequency on such measurements. Similarly, it has been shown that low modulation frequencies penetrate more deeply than high frequencies.^30^ This spatial discrimination may be another benefit to broadband frequency sweeps.

Additionally, there are other limitations of this study which should be kept in mind when interpreting the results. Although we attempted to accurately reproduce the noise characteristics of each system, several aspects that affect noise were not considered in this analysis. The first is that only data from the 850-nm laser for both systems was shown. Each laser had its own specific noise level, though they all followed a similar trend. Finally, this analysis assumes that the amplitude and phase noise at different frequencies was uncorrelated. In fact, amplitudes, especially at low modulation frequencies, were relatively highly correlated with one another (a Pearson correlation coefficient of about 0.4). Incorporating correlation between frequencies would increase the error when multiple frequencies were used because each additional frequency would provide less new information. Overall, the presence of correlations would enhance the advantage of repeated measurement at a single frequency over a single broadband measurement.

## 5 Conclusion

This study addressed the issue of FD-DOS modulation frequency bandwidth using experimentally derived noise models for two FD-DOS systems. A major result of this analysis was that repeated measurements at a single low-frequency yielded essentially identical errors as broadband frequency sweeps using a light transport model of homogeneous media. This has important implications for FD-DOS instrument design going forward, as single-frequency devices are generally less complex and allow for increased acquisition speeds.

An additional important result was the demonstration of the importance of careful selection and optimization of the OP inverse model, which contributes to overall system errors. When an optimized iterative method is used, the extracted error can be essentially eliminated for noise-free simulated data, which allows accurate extraction in nearly all conditions. As the optimized method can be slow, we also developed a new LUT approach suitable for use with multiple frequencies. This method is capable of very high-speed inversions when few frequencies are used and may be useful for calculation and display of OPs and chromophore concentration in real time.

## Code, Data, and Materials Availability

All code and data used in the production of this manuscript is available on github: https://github.com/BU-BOTLab/frequencySelection.
