# Supplementary material for: Modulation frequency selection and efficient look-up table inversion for frequency domain diffuse optical spectroscopy
Source: J Biomed Opt. 2021 Mar 25;26(3):036007. doi: 10.1117/1.JBO.26.3.036007 (PMC7992233; doi:10.1117/1.JBO.26.3.036007)
Supplement: Supplementary file 1 [file JBO_026_036007_SD001.pdf]

Supplementary Material for:

Modulation frequency selection and efficient look-up table inversion for Frequency Domain Diffuse Optical Spectroscopy

Matthew B. Applegate[a], Carlos A. Gómez[a], Darren Roblyer[a,*]

[a]Department of Biomedical Engineering, Boston University, 44 Cummington Mall, Boston, MA 02215, USA

Fig. S1 shows the error landscapes from the right column of Fig. 2 in the main body of the paper split into absorption error (left) and reduced scattering error (right) displayed as percent error. Fig. S2 shows the error landscapes from Fig. 8 of the main paper split into absorption error (top) and reduced scattering error (bottom) displayed as percent error. For the simulated, noise-free data in Fig. S1, the absorption error tends to be higher than the scattering error, while for simulated noisy data in Fig. S2 the error is more equally distributed between absorption and scattering.

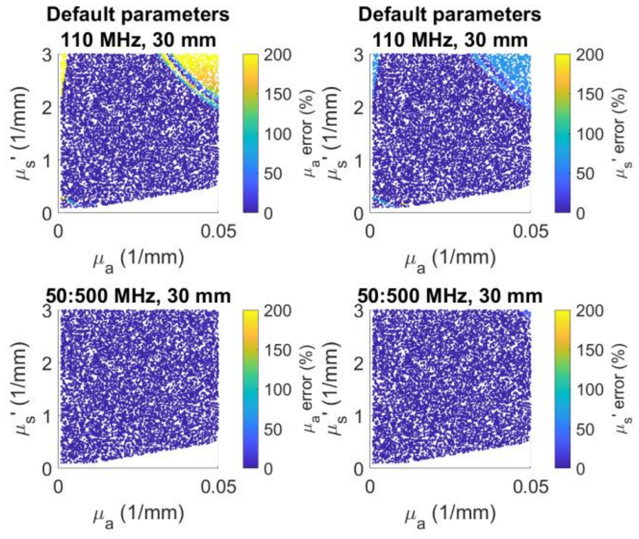

Fig. S1: Error landscape plots for noise-free data processed using the default parametrs shown in percent error for absorption (left) and scattering (right) respectively

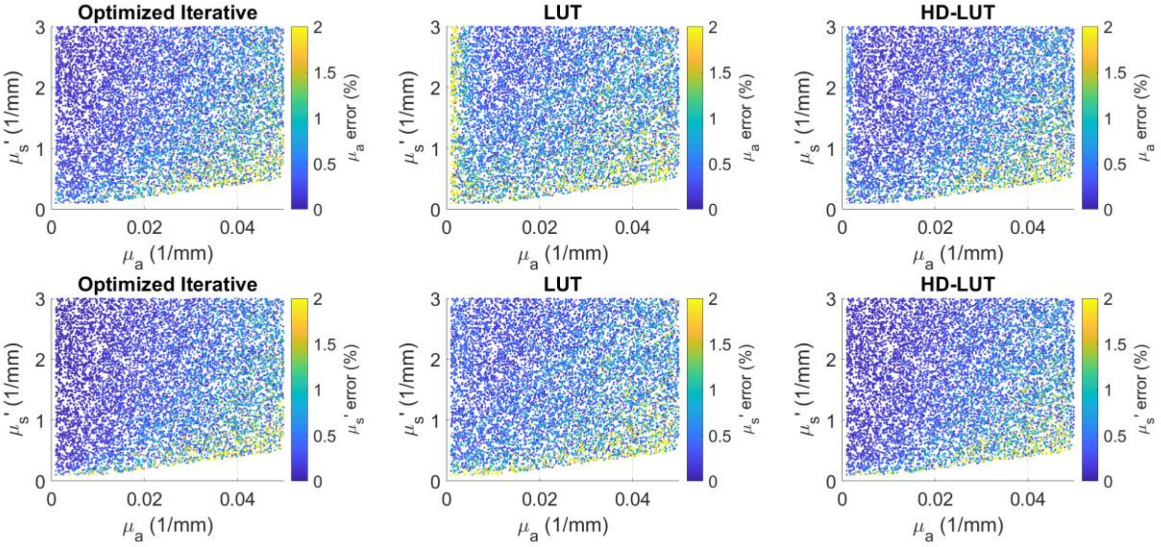

Fig. S2 Error landscape plots comparing the error between the iterative and LUT methods shown in percent error for absorption (top) and scattering (bottom) respectively
